# Disability and premorbid adjustment in schizophrenia: A retrospective analysis

**DOI:** 10.4102/sajpsychiatry.v28i0.1853

**Published:** 2022-12-19

**Authors:** Omokehinde O. Fakorede, Adegboyega Ogunwale, Akinwande O. Akinhanmi

**Affiliations:** 1 Department of Clinical Services, Neuropsychiatric Hospital, Aro, Abeokuta, Nigeria; 2 Department of Mental Health and Behavioural Medicine, Federal Medical Centre, Abeokuta, Nigeria

**Keywords:** psychosis, limitation, pre-illness functioning, correlation, Nigeria

## Abstract

**Background:**

Schizophrenia is highly disabling. Though efforts at genetic mapping to identify those at risk of the illness have been promising but same cannot be said about predicting its associated disability before illness-onset (i.e., during premorbid phase). It is envisaged that Schizophrenia-related disability may be ameliorated if premorbid clinical markers are adequately predictive enough to identify those at risk and worked upon them.

**Aim:**

This study aimed to determine whether there is a relationship between schizophrenia-related disability and premorbid adjustment.

**Setting:**

This cross-sectional study was conducted at the out-patient clinic of the Neuropsychiatric Hospital, Aro, Abeokuta, Ogun State, Nigeria.

**Methods:**

Three hundred patients with the diagnosis of schizophrenia and within the age range of 18–64 years were eligible for the study.

**Results:**

Mean age of the participants was 41.9 ± 10.05 years with a slight female dominance (50.7%). Spearman’s correlation revealed a direct correlation between disability and premorbid adjustment, albeit rather weak (*r*_s_ = 0.130, *p* = 0.025).

**Conclusion:**

Pre-diagnostic factors such as premorbid functioning may play a role in the subsequent functioning of an individual post-diagnosis. Other research efforts may focus on yet-to-be identified premorbid factors that may be targets of prevention to reduce disability in schizophrenia.

**Contribution:**

This research serves as a pioneer work on disability and premorbid adjustment and has provided a template for the early identification of those at risk of schizophrenia by providing an intervention opportunity at the premorbid stage.

## Introduction

Studies on disability associated with schizophrenia have commanded the attention of authors for over four decades.^1,2,3,4,5^ Research has shown that the prevalence of disability among patients with schizophrenia is high,^6^ and higher than in the general population.^5^ Prevalence rates of 78.0% – 96.7% have been typically reported by various authors.^4,5,6^ Disability studies among patients with mental illness have also revealed that more of those with schizophrenia have disability and even of a higher severity than others.^7^ Furthermore, a study has demonstrated that disability is mostly experienced in the domains of societal interaction and participation.^8^ Emotional blunting, apathy and stigma have been identified as major contributors to this finding.^9,10^ Various anatomical locations such as the dorsolateral prefrontal cortex and the medial temporal lobe^11,12^ have also been implicated in the pathophysiology of this construct among patients with schizophrenia.

The role of some post-diagnostic factors in attenuating disability has been elucidated in literature. These include the use of newer generation anti-psychotics,^11^ medication adherence,^12^ appropriate caregiver’s expressed emotion,^13^ lower levels of stigma,^14^ good family support^15^ and being in employment.^15^ Similarly, quite a number of pre-diagnostic factors have also been explored. For instance, genetics,^16^ obstetric events^17^ childhood trauma,^18^ upbringing,^19^ migration,^20^ and even premorbid personality^21^ have all been investigated.

However, an important ‘global’ assessment of an individual’s level of functioning before the development of the illness – *Premorbid Adjustment* – appears to have been overlooked. Premorbid adjustment, an important contributor to self-esteem,^22^ describes the level of functioning of an individual from birth to 1 year prior to the onset of psychotic illness. Apart from ascertaining the quality of life of an individual, sacrosanct aspects of life are also covered during the assessment of premorbid adjustment – sociability, withdrawal, peer relationships, scholastic performance, school adaptation, socio-sexual interactions, school or job stability, independent living, societal or leadership role performance, leisure and average energy level. Opinions of authors on the relationship between premorbid adjustment and disability are diverse. Tsang et al.^23^ showed that being employed prior to the onset of a psychiatric illness reduces the likelihood of being occupationally disabled post-diagnosis, but Schoeyen et al.^24^ found no relationship between premorbid adjustment and occupational outcome among Norwegian patients with bipolar disorder. In relation to schizophrenia, Ayesa-Arriola et al.^25^ found that premorbid functioning in the social domain has a significant association with social disability over a three year follow-up and it is the strongest predictor of outcome. In the same vein, Ojeda et al.^26^ narrated a predictive relationship between poor premorbid adjustment and outcome, while Petersen et al.^27^ posited a direct relationship with full ‘recovery’, both among groups of patients with schizophrenia.

Though there is a great dearth of research on this subject matter in Nigeria and Africa, it is evident that the studies referred to earlier have investigated only certain aspects of premorbid adjustment (e.g., social^25^ and occupational adjustment)^23^ with key findings related to a few specific outcomes (occupational functioning, recovery). Some others^6^ have comprehensively assessed various domains of premorbid state and investigated their relationship with one of the few outcomes. Nigeria has no white paper serving as guidelines for the management of schizophrenia and neither does the country have annual concrete plans to cater for disability linked to schizophrenia or other mental illnesses. Clearly, a more global assessment of premorbid functioning in relation to relevant comprehensive and multi-faceted outcomes such as disability is needed. It is on this background that the study was conceptualized. The objective of this study was to explore the relationship, if any, between on-going schizophrenia-related disability and the level of functioning prior to the onset of illness.

## Research methods and design

The study was a cross-sectional study conducted at the outpatient clinic of the Neuropsychiatric Hospital, Ogun State, Nigeria. The study centre provides both in-patient and out-patient services to patients with various psychiatric and neurological conditions.

Participants were consenting patients with an International Statistical Classification of Disease and Related Health Problems (ICD-10)^28^ diagnosis of schizophrenia and who were within 18–64 years age group (exclusion criteria includes having a diagnosis of dementia, learning disability, obvious physical handicap or any chronic disabling condition). The study size was obtained using the Cochran minimum sample size formula^29^ of *n* = Z^2^pq/d^2^ to yield a sample size of 300. The sample size was derived from using 78% as *p*, being the prevalence of social disability among patients with schizophrenia attending the out-patient clinic of the same hospital in the year 1999.^6^ The initial figure obtained was 264 but with correction for true sample size, possible refusal and non-response, the figure increased to 294 which was rounded up to 300:


$$n=\frac{Z^{2}\;pq}{d^{2}}$$ [Eqn 1]

where:

*n* = the desired sample size when the population is > 10 000

*Z* = the standard normal deviate, 1.96 at 95% confidence level

*p* = the proportion of social disability among outpatients with schizophrenia, 78%

*q* = 1.0 – *p* = 0.22

*d* = degree of accuracy desired usually set at 0.05.

Therefore:


$$\begin{array}{l} n=\frac{1.96\times 1.96\times 0.78\times 0.22}{0.05\times 0.05}\\ n=263.68 \end{array}$$ [Eqn 2]

However, because the study population is below 10 000, the true sample size (*n*_*f*_) was estimated as:


$$n_{f}=\frac{n}{1+\frac{\left(n\right)}{N}}$$ [Eqn 3]

where:

*n*_*f*_ = the desired sample size when the study population is less than 10 000

*n* = the desired sample size when the study population is more than 10 000, that is, 264

*N* = the estimated study population which is 9417, the population of outpatients within the age of 18–64 years and with the diagnosis of schizophrenia in the year 2010.

Therefore:


$$\begin{array}{l} n_{f}=\frac{264}{1+\frac{264}{9417}}\\ n_{f}=256 \end{array}$$ [Eqn 4]

This was then oversampled by 15% to account for refusal and non-response, which gave a total of 294.4–300.

This work is part of a larger study titled ‘A study of disability among outpatients with Schizophrenia attending the Neuropsychiatric Hospital, Aro, Abeokuta’. Patients who met the study criteria and gave their informed consent were recruited through convenient sampling method. Each of the participant was interviewed with a socio-demographic proforma, World Health Organization Disability Assessment Schedule, version 2.0 (WHODAS 2.0)^30^ and the Premorbid Adjustment Scale (PAS).^31^ For the WHODAS 2.0, the range of obtainable summary score is 0–100. Applying the population norms for the item response theory (IRT)-based scoring for this 36-item version, a summary score of 0, 1–41 and > 41 correlates with *no, mild or moderate* and *severe or extreme disability,* respectively (WHO, 2010). On the other hand, the premorbid adjustment scores were categorized as progressive decline, stable-poor or stable-good. The progressive decline group refers to those who consistently had worsening premorbid adjustment scale (PAS) scores from childhood over the subsequent premorbid periods with an equivalent of at least 2-point change across the four periods. The stable-poor and stable-good groups refer to those who show a pattern of stability across the premorbid period. The median value of the PAS score of the patients in the latter two categories was used to dichotomize them into either group. Scores below the median were grouped as stable-good while those above as stable-poor.^32^ Each interview lasted about 30 min on the average.

The primary outcome measures were schizophrenia-related disability, premorbid adjustment and the relationship between the two.

Data were analysed with the use of the IBM Statistical Package for Social Sciences (SPSS), version 23.^33^ Continuous data were presented in range, means (with standard deviation) and median (with interquartile range), while nominal variables of categorical data were expressed as percentages. Spearman’s rank-order correlation was run to determine the relationship between disability and premorbid adjustment scores.

### Ethical considerations

The authors assert that all procedures contributing to this work complied with the ethical standards of the relevant national and institutional committees on human experimentation and with the Helsinki Declaration of 1975, as revised in 2008. All procedures involving human subjects and/or patients were approved by Neuropsychiatric Hospital Aro Research Ethics Committee (Approval Number – PR001/130). Written informed consent was obtained from all subjects and/or patients.

## Results

**FIGURE 1:**
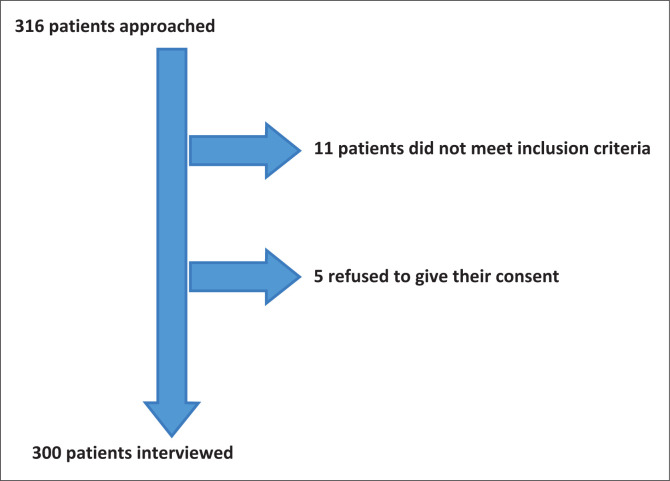
Flow diagram illustrating the recruitment process.

A total of 316 were invited to participate. Eleven patients did not meet the diagnostic criteria for schizophrenia and five patients refused to give their consent. Therefore, 300 patients were recruited for the study (Figure 1).

Table 1 shows that the female participants were slightly more (50.7%) and the mean age of the study sample was 41.9 ± 10.05 years. Almost half of the respondents (43.3%) had completed secondary education while about two-thirds (64.0%) were meaningfully engaged either as student, apprentice or professionals in their fields.

**TABLE 1:** Socio-demographic profile of participants (*N* = 300).

Variables	*n*	%
**Gender**		
Male	148	49.3
Female	152	50.7
**Age**		
20–29	29	9.7
30–39	107	35.6
40–49	89	29.7
50–59	60	20.0
60–69	15	5.0
Mean ± s.d.	28.4 ± 8.5	9.7
**Employment status**		
Unemployed	100	33.3
Student or apprentice	24	8.0
Employed	168	56.0
Retired	8	2.7
**Highest educational status**		
None	16	5.3
Primary	56	18.7
Secondary	130	43.3
Tertiary and postgraduate	98	32.7

Note: Mean ± s.d. = 28.4 ± 8.5 and 9.7%

s.d., standard deviation.

Table 2 highlights the sample’s mean age at illness onset and duration of illness – 28.4 ± 8.5 and 13.5 ± 8.6 years respectively.

**TABLE 2:** Clinical profile of the participants.

Variables	Mean	s.d.
Age at illness onset (years)	28.4	8.5
Total illness duration (years)	13.5	8.6
DUP (weeks)	187	155.1
Number of episodes	2.6	1.0
Number of previous hospitalization	0.9	1.0

DUP, duration of untreated psychosis.

**FIGURE 2:**
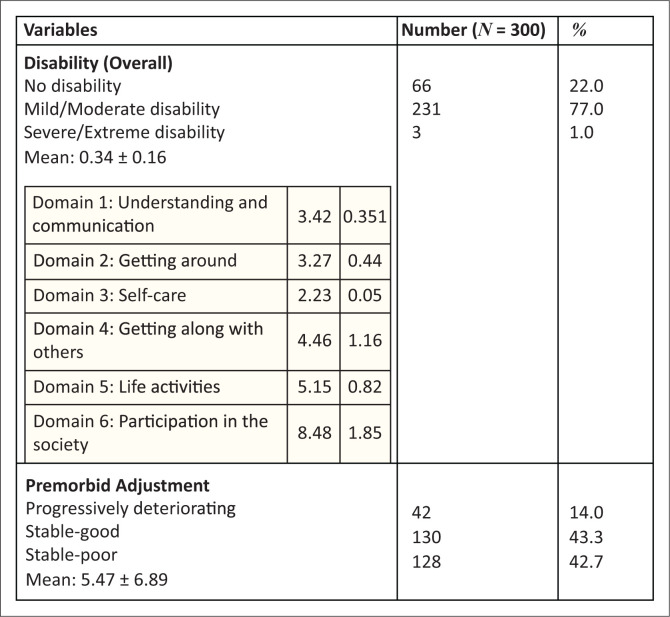
Prevalence of disability and premorbid adjustment (outcomes).

Details of disability (overall and domain) and premorbid adjustment scores are shown in Figure 2. The median (IQR) score of the subsample, after excluding those with a progressively deteriorating course, was 0.28 (0.05) and this was used to dichotomize the remaining PAS scores into stable good and stable poor groups. The median (interquartile range [IQR]) disability and premorbid adjustment score were 0.29 (0.16) and 3.33 (6.44), respectively. The disability and premorbid adjustment scores of both male and female participants were not statistically significant – *t*(298) = −1.74, *p* = 0.083; *t*(298) = −2.77, *p* = 0.006.

**FIGURE 3:**
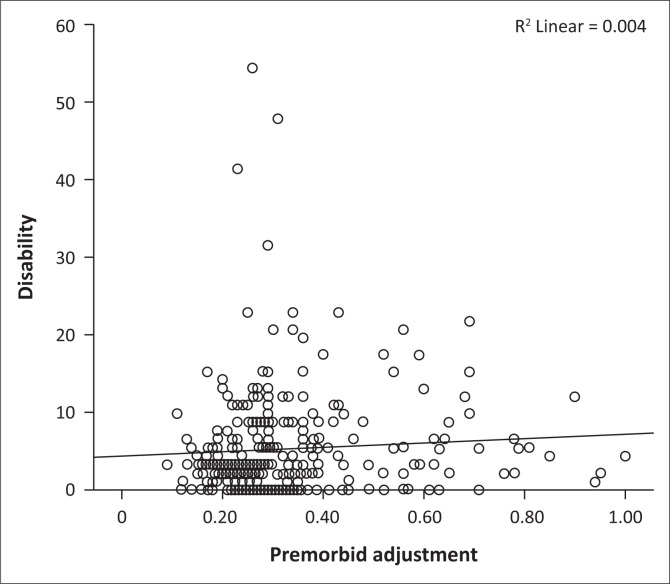
Scatter diagram showing the relationship between disability and premorbid adjustment.

Figure 3 shows that there is a fairly linear and direct relationship between disability and premorbid adjustment. A Spearman’s rank-order correlation between the duo (Table 3) yielded a weak positive correlation which was statistically significant (*r*_s_ = 0.130, *p* = 0.025).

**TABLE 3:** Correlation between disability and premorbid adjustment.

Spearman’s rho	Overall PAS score	Overall disability score
**Disability**		
Correlation coefficient	1.00	0.13
Sig (2-tailed)	-	0.03
***N***	300	300
**Premorbid adjustment**		
Correlation coefficient	0.13	1.00
Sig (2-tailed)	0.03	-
***N***	300	300

PAS, premorbid adjustment scale; Sig, significance.

## Discussion

Investigating the relationship between premorbid adjustment and disability is quite novel. This study has brought to light the role of premorbid abilities in the continuum of normality to post-abnormality as pertaining to the outcome of schizophrenia.

Although the sample largely consists of young to middle age groups, about half adjusted poorly premorbidly and a higher number had schizophrenia-related disability.

Three-quarters of the study sample reporting disability means that about 7 out of 10 patients with schizophrenia experience some difficulty with executive functions, mobility, self-care, relationships and functioning at work or school. This is in line with the findings of other authors. Gureje and Bamidele^6^ reported a disability rate of 78% while Ali^34^ as well as Wiersma et al.^4^ reported a figure of 83% among patients with the same illness. Even rates as high as 100% have been reported by some other authors.^1^ All these support the fact that the illness itself confers some difficulty in carrying out age- and gender- appropriate tasks.^1,35^

Different underlying mechanisms such as motivation,^36^ functional capacity^9^ and neurocognitive deficits in the dorsolateral prefrontal cortex and the medial temporal lobe^37^ have been identified as being responsible for disability in schizophrenia but the most consistent is the latter of the three.^10,32^

Rising premorbid adjustment scores indicate worsening premorbid states because a score of 0 means the healthiest state while the maximum score of 6 depicts the worst premorbid state. On the other hand, rising disability score is synonymous with worsening disability state. This study demonstrated a direct but weak correlation between disability and premorbid adjustment – that is individuals with a high level of disability must have had a poor premorbid adjustment.

In a restrictive sense, Bailer et al.^38^ stated that poor social premorbid adjustment, more often than not, implies social disability. Similarly, Ayesa-Arriola et al.^25^ who broadened the outcome of interest to functional disability found that poor social premorbid adjustment was one of the three strongest predictors for functional disability over a three year period. It is important to note that the functional outcome investigated in the latter study entailed the use of Disability Assessment Schedule which also measures social relationships.

Focusing on only an aspect of premorbid functioning may be a common limitation of these studies, but its major outcome – social disability – is an area where patients with schizophrenia are mostly disabled. It may then be inferred that social dysfunction in individuals may be a pointer to evolving schizophrenia.^39,40,41,42^

Firstly, the ‘weakness’ of this correlation may be because of the skewness of having a relatively smaller percentage without disability. Secondly, another perspective to this may be that other correlates, in addition to poor premorbid adjustment, must be present for disability to be fully expressed. These variables may have been present in only a minority of this sample population or may not have been investigated in this study. A relevant case in point is the contributory role of depression to disability in patients with schizophrenia.^43^ According to the authors, the presence of depression contributes largely to the expression of disability in the same patient. Thirdly, poor premorbid adjustment may not be a *sine qua* to disability. In other words, a poorly (premorbidly) adjusted patient with schizophrenia may or may not experience difficulties in any domain of life. For such an individual, not experiencing any difficulty may be because of the presence of positive prognostic factors such as strong social network.^44,45^ Social networking is prevalent in non-Western societies, including Nigeria, which thrives on strong family ties and community support.^46^ Patients with mental illnesses, including schizophrenia, usually benefit from informal caregiving provided by their families^37^ and this may explain why an individual with poor premorbid functioning may not progress to disability. This may be worthy for further exploration in order to add to the existing knowledge on schizophrenia for the Nigerian context.

An important finding from literature is the relationship between premorbid adjustment and full recovery in schizophrenia. Torgalsbøen^47^ found that good premorbid adjustment slightly predicted full recovery. This may not be unconnected with the former’s role as an index of cognitive reserve and its predictive power for global cognitive improvement.^48^

Although the sample sizes of these studies were modest (50 and 79) and these findings^47,48^ appear fairly reliable. Based on these, it may be inferred that adequate intervention at the premorbid phase may yield good recovery and as a result, reduce the prevalence rates of treatment-resistant variants of the illness. No doubt, schizophrenia is disabling, but efforts to reduce the incidence of relapses and ensure full recovery may reform the public’s perception of the illness.

Apart from utilizing a large sample size, this research serves as a pioneer work on disability and premorbid adjustment. It is also an eye-opener on the severity of schizophrenia-related disability in this current dispensation of new generation antipsychotics in Nigeria. This research has provided a template for the early identification of those at risk of schizophrenia by providing an intervention opportunity at the premorbid stage. By so doing, the incidence of those with schizophrenia as well as associated disability may reduce. Overall, costs incurred in managing disability or the economic loss in neglecting it will reduce drastically. Findings from this work may also provide healthcare managers as well as policy makers with a template for formulating better mental healthcare plans and monitoring its implementation.

Recall bias in the premorbid account given by patients is a limitation but efforts were made to reduce this by corroborating information from more than one relative. Current disability (from other non-psychiatric factors) being experienced by patients may also colour/alter their report of premorbid functioning.

## Conclusion

Premorbid adjustment appears to have a positive, albeit weak relationship with disability in schizophrenia. The plausible role of other determinants to the weak association between both constructs should not be ignored. Individuals with poor social relationships and/or functioning may just be at a pre-psychotic phase, necessitating a structured preventative approach to prevent the onset of schizophrenia or better still mitigate against the development of its consequential social disability. In addition, patients with a diagnosis of schizophrenia need to be followed up from the onset, and for a long period to determine if and when the disability resurfaces. This also gives further support to the need for national registers for psychiatric conditions in Nigeria which will make contact and follow-up easier.
